# Tolerability and efficacy of long-term treatment with daptomycin, ceftazidime and colistin in a patient with a polymicrobial, multidrug-resistant prosthetic joint reinfection: a case report

**DOI:** 10.1186/1752-1947-8-186

**Published:** 2014-06-12

**Authors:** Maria Bruna Pasticci, Paolo Di Filippo, Leonella Pasqualini, Antonella Mencacci, Carlo Pallotto, Lisa Malincarne, Franco Baldelli

**Affiliations:** 1 Infectious Disease Section, Department of Experimental Medicine and Biochemical Sciences, University of Perugia, 06100 Perugia, Italy; 2 Orthopedic Department, Ospedale Santa Maria, Terni, Italy; 3 Internal Medicine Section, Department of Medical Sciences, University of Perugia, Perugia, Italy; 4 Microbiology Section, Department of Experimental Medicine and Biochemical Sciences, University of Perugia, Perugia, Italy

## Abstract

**Introduction:**

Prosthetic joint infections are severe complications of joint implants. Further complications arise when polymicrobial and/or multidrug-resistant microorganisms are involved. Currently, there are limited data on the management of these infections and on the tolerability of long-term treatment with daptomycin, ceftazidime and colistin.

**Case presentation:**

A 55-year-old Caucasian woman who had a right hip prosthesis removed 1 year prior because of infection was admitted for prosthesis reimplantation. On admission at our hospital, anamnesis regarding etiology and management of prosthesis infection was not available. On clinical, laboratory findings and imaging studies infection was not suspected. A hip prosthesis was reimplanted. At surgery, histopathological and microbiological investigations were not taken. Three weeks after reimplantation, surgical site infection due to *Enterobacter cloacae* was diagnosed and oral ciprofloxacin was prescribed. Four days later, a periprosthesis fluid collection was evidenced and a percutaneous needle aspirate grew *Staphylococcus epidermidis* and *S. haemolyticus. Enterobacter* genome was also detected from the same sample. Teicoplanin and meropenem were added to ciprofloxacin without clinical improvement. Moreover, acetabular cup dislocation was documented. She underwent prosthesis explantation, debridement, and positioning of an antimicrobial mixed spacer. From the intraoperatory cultures *S. epidermidis* and *Acinetobacter baumannii* were grown. Daptomycin, ceftazidime, colistin and rifampin were administered. Four days later, rifampin was stopped due to a suspected liver toxicity. While undergoing therapy she presented recurrent episodes of wound dehiscence and on the 22nd week of treatment a further surgical debridement was performed, upon which the spacer was removed. At this time, intraoperative cultures resulted negative. Three months later, after a total of 8 months, antimicrobials were interrupted. Subsequently, a femoral transcondylar traction was positioned, and 3 weeks later a new prosthesis was reimplanted. At over 1 year after reimplantation she is well.

**Conclusions:**

Our findings suggest that microbiologic investigations are mandatory even when prosthetic joint infection is not suspected. Molecular methods for identification of microorganisms can be used in addition to conventional cultures especially when patients are under antibiotic treatment. Daptomycin, ceftazidime and colistin can be administered for several months without side effects. Guidelines specifically addressing the diagnosis and the management of polymicrobial, multidrug-resistant prosthetic joint infections need to be developed.

## Introduction

Prosthetic joint infection (PJI) is one of the most severe complications of joint replacement [1]. Although this is a rare event [1], the overall burden is high as a consequence of an increased number of implanted prosthesis in the aging population, an increased number of patients with risk factors for infection and improved methods to detect these infections. PJIs are associated with high morbidity, a need of complex treatment, prolonged hospitalization and substantial healthcare costs. Moreover, PJIs can lead to impaired functioning or even permanent disability [1,2]. Further complications arise when polymicrobial, multi-drug-resistant (MDR) [3,4] or difficult to treat microorganisms are involved [5-10].

Here, a complicated case of PJ reinfection is reported.

## Case presentation

**Figure 1:**
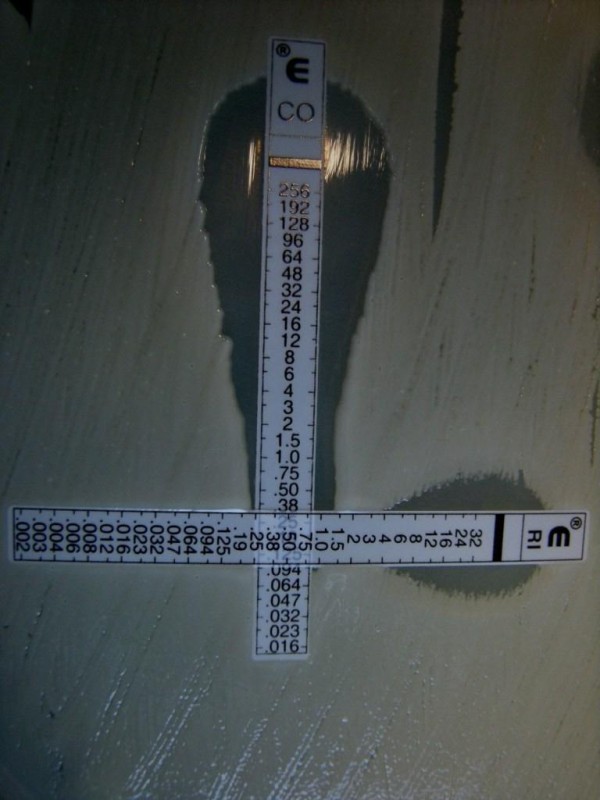
***Acinetobacter baumannii*: E-test, interaction between colistin and rifampin.** Fractional inhibitory concentration=1.37 [13].

**Figure 2:**
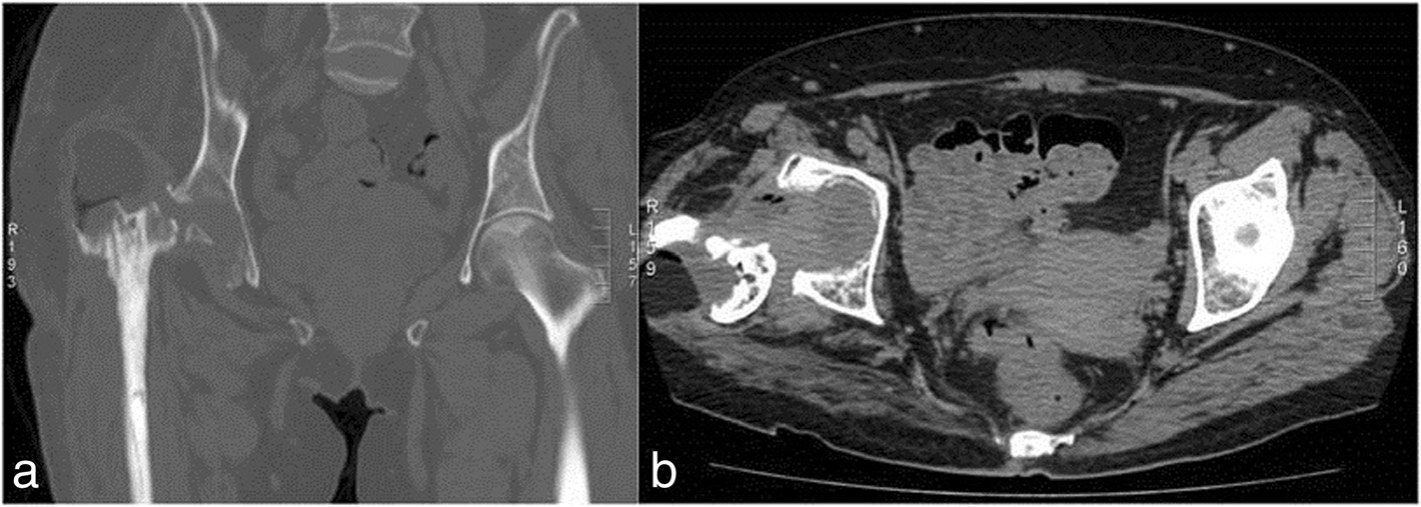
**Computed tomography. a)** Coronal and **b)** axial images showing fluid around the spacer, femoral inflammation, and spacer dislocation.

A 55-year-old Caucasian woman was admitted for a right hip prosthesis reimplantation. The first arthroplasty was performed 3 years earlier due to hip osteoarthritis. However, after 2 years her prosthesis had been explanted and a spacer positioned due to infection. After 1 month the spacer had also been removed. No further data on microbiological results and medical treatment were available at the time of her admission at our hospital. On admission, she was complaining of pain and was not taking antibiotic therapy. Her erythrocyte sedimentation rate (ESR) was 35mm 1°hour, her white blood cell count and differential were normal, C-reactive protein (C-RP) was not available and a leukocyte scan resulted normal. She underwent hip reimplantation. At surgery, histopathological and microbiological investigations were not taken. After arthroplasty, she was discharged. Two weeks later she was seen as an out-patient complaining of hip pain, motion impairment and dehiscence of the wound. *Enterobacter cloacae* was grown from the wound exudate. The isolate was an extended spectrum β-lactamase producer, resistant to gentamicin, and susceptible to ciprofloxacin, imipenem, and colistin, according to Clinical and Laboratory Standards Institute (CLSI) breakpoints [11]. She was started on a treatment with oral ciprofloxacin 500mg twice per day. Four days later, ultrasound evidenced periprosthesis fluid collection. *Staphylococcus epidermidis* and *S. haemolyticus* were cultured from the needle aspiration. Both coagulase-negative staphylococci (CoNS) isolates resulted oxacillin and ciprofloxacin resistant, teicoplanin susceptible [11]. The same sample was also examined with the commercial real-time polymerase chain reaction-based system, SeptiFast (Roche Molecular Diagnostics, Mannheim, Germany) which detected *E. cloacae/aerogenes* and CoNS genomes. At this point, the patient was readmitted. Teicoplanin intravenous 400mg per day and meropenem intravenous 2g three times per day (patient’s weight was 68kg) were added to ciprofloxacin without clinical improvement. After 2 weeks, *E. cloacae* with the same susceptibility pattern of the previous isolate and *Acinetobacter baumannii* were grown from the aspirated synovial fluid of her hip. *A. baumannii* isolate resistant to aztreonam, cefepime, cefotaxime, ciprofloxacin, imipenem, fosfomycin, gentamicin, and trimethoprim/sulfamethoxazole, but susceptible to ceftazidime and colistin [11] was obtained. Tigecycline minimal inhibitory concentration was 1.5mg/L, however, no breakpoints were available for this antimicrobial agent against *A. baumannii*, according to CLSI [11]. At this point, a standard radiograph evidenced acetabular cup dislocation, therefore her prosthesis was removed. During surgery, extensive debridement was performed and a spacer with vancomycin and gentamicin was inserted. From the periprosthesis tissue samples, and her prosthesis, *S. epidermidis* and *A. baumannii* were identified while from the synovial fluid only *S. epidermidis* was isolated. Susceptibility patterns of *A. baumannii* isolates did not differ. The susceptibility pattern of the two latter *S. epidermidis* isolates was different from that of the previous one in respect to erythromycin, clindamycin, and trimethoprim/sulfamethoxazole. Ceftazidime was added to the pre-existing therapy. When *in vitro* antimicrobial susceptibility [11] with synergism results (Figure 1) [12,13] were made available, therapy was modified as follows: daptomycin intravenous 500mg per day, ceftazidime intravenous 2g three times per day, colistin intravenous 3 million units three times per day, and rifampin 600mg daily administered orally. Four days later, rifampin was stopped due to a suspected liver toxicity. Overall, her condition improved despite recurrent episodes of wound dehiscence and purulence. After almost 12 weeks of antimicrobial treatment, she was accepted into a protected residence where she continued to undergo treatment of intravenous antimicrobial therapy with daptomycin 500mg per day, ceftazidime 2g three times per day, and colistin 3 million units three times per day. One month later, a computed tomography (CT) scan of her hip showed liquid around the spacer and femur inflammatory reaction (Figure 2). Two weeks later, another dehiscence of the wound manifested. She was readmitted for an ulterior debridement and this time the spacer was also removed. Prior to surgery antimicrobial therapy was not interrupted. Intraoperative microbiological investigations resulted negative including the molecular SeptiFast test. After surgery she returned to the protected residence and continued the same antimicrobial therapy. Three weeks later, colistin was reduced from 3 to 2 million units intravenous per day every 8 hours. After a total of 8 months, all antimicrobials were stopped. During the entire period antimicrobial therapy was administered, she was clinically monitored and, every 10 days, ESR, C-RP, blood count, creatine phosphokinase, liver and kidney function tests and electrolytes were obtained. No side effects were observed during treatment. When antimicrobials were discontinued, a further CT of her hip evidenced dislocation of her femur and inflammatory tissue surrounding her femur and the acetabular cavity. During the following 3-month period, she did not manifest clinical evidence of infection, and her ESR and C-RP were normal; then she was readmitted to another hospital to be reimplanted. A femoral transcondylar traction was first positioned for over 3 weeks in order to extend her muscles, thereafter a third prosthesis was implanted. At surgery, there was no evidence of purulence; however, there was necrotic tissue which underwent debridement. After several samples were collected for microbiologic investigations, she was administered daptomycin intravenous 500mg per day, ceftazidime intravenous 6g per day, and colistin intravenous 2 million units three times per day until microbiological results, including the SeptiFast test, were reported negative. For more than a year since her third prosthesis was reimplanted, she has been asymptomatic and has regained motility.

## Discussion

There are several guidelines on the diagnosis and the management of PJIs; however, polymicrobial, MDR and difficult to treat PJIs [5,6] have not been thoroughly addressed.

This article reports a polymicrobial, MDR prosthesis reinfection successfully treated with a two-stage long interval reimplant and prolonged antimicrobial therapy.

In our case, *E. cloacae*, *A. baumannii* and CoNS were the more probable etiologies of the infection. In fact, all the microorganisms, even in different combinations and at different times, were identified from multiple intraoperative tissues, prosthesis, and periprosthesis and synovial fluid aspirates [14-16]. It is impossible to state if any of the isolated microorganisms had already been present in a nonreplicating phase in the involved articulation at the time of the first reimplantation at our hospital. Nonperforming intraoperative histopathological and microbiological examinations can lead to missed cases of prosthesis infections [2,5,14-16]. Improper sample collection, small colony variants [7,8], antimicrobial therapy before or during surgery and a lack of sonication [17-19] can also hinder diagnosis and etiology of PJIs [1,2,5,13-20]. Small colony variants as well can lead to problems in microorganism identification and antimicrobial susceptibility results [7,8]. However, in our case, it was not possible to verify if the observed differences for CoNS in respect to susceptibility and identification results were due to small colony variants.

The SeptiFast test has been commercialized for the diagnosis of sepsis from peripheral blood samples and identifies 25 different common pathogens [21]. This test has been suggested to have a potentiality in detecting pathogens also from specimens other than blood, like sonicated fluids from removed prosthesis and synovial fluids [19,22]. Its sensitivity was better than that of culture in patients under antibiotic treatment [22]. In our case *Enterobacter cloacae*/*aerogenes* and CoNS genomes were detected with the SeptiFast test in the aspirated periprosthetic fluid, and contemporary cultures grew *S. epidermidis* and *S. haemolyticus.* At that time, the patient had been diagnosed surgical site infection and was on oral ciprofloxacin, active *in vitro* against *E. cloacae* but not against CoNS. The clinical significance of deoxyribonucleic acid (DNA) in the blood of septic patients in the absence of microorganism growth is still not optimally defined [21] and there is limited data on other biological samples. However, in our case, the fact that, 8 days later, *E. cloacae* was isolated from the aspirated synovial fluid points to the clinical relevance of the molecular results. The absence of bacterial DNA and viable microorganisms in intraoperative samples at the time the prosthesis was implanted for the third time was used to further support the absence of residual infection. Culture has the ability to identify microorganisms not included in the SeptiFast panel and to allow susceptibility testing. Thus, it is reasonable to use a molecular test in adjunct to culture in patients with PJIs under antibiotic therapy.

Regarding the most effective surgical strategy for PJIs and the best time of prosthesis reimplantation, the decision depends on the operating orthopedic surgeon, medical specialists, and the patient [16]. Concerning the use of spacers, some researchers recommend avoiding cement spacers when infections are due to MDR or difficult to treat microorganisms such as quinolone-resistant and rifampin-resistant staphylococci, quinolone-resistant Gram-negative microorganisms, small colony variants, or fungi [5-8,16,20]. However, other researchers suggest avoiding external fixations in the presence of bone infection, but instead perform debridement repeatedly and change spacers as necessary. Furthermore, antimicrobial impregnated spacers, either premixed or prepared by the surgeons, can cause systemic toxicity [16]. The optimal timing for prosthesis reimplantation is another issue lacking controlled randomized studies to support a specific recommendation [16]. In our patient, initially, a cement spacer was positioned, then, it was removed and for a 3-month interval our patient was left without a spacer, continuing intravenous antimicrobial therapy. Then, after a long interval without antimicrobials, clinical signs of infection and normal ESR and C-RP results, she was readmitted for reimplantation. Overall, in our patient there was a delay of 1 year between prosthesis explant and reimplantation, due to the fact that: 1) clinically, the infection was not fully controlled until the spacer was left in place, 2) the patient had undergone hip prosthesis surgery twice and was at high risk for further infective complications.

In our case, recovery from infection was obtained with surgical therapy and a combination of antimicrobial treatment with daptomycin, ceftazidime, and colistin that were finally efficacious and well tolerated. It is possible that initial antibiotic therapy with teicoplanin, ceftazidime and ciprofloxacin was not completely effective due to biofilm infection, suboptimal dosing of teicoplanin, and resulting CoNS ciprofloxacin resistance.

## Conclusions

Our findings suggest that microbiologic investigations are mandatory even when PJI is not suspected on clinical and laboratory findings and imaging studies. Molecular methods for identification of microorganisms can be used in addition to conventional cultures in patients with PJIs especially when they are under antibiotic treatment. Daptomycin, ceftazidime and colistin can be administered for several months without side effects. Guidelines specifically addressing the diagnosis and the management of polymicrobial, MDR PJIs need to be developed.

## Consent

Written informed consent was obtained from the patient for publication of this case report and any accompanying images. A copy of the written consent is available for review by the Editor-in-Chief of this journal.

## Competing interests

The authors declare that they have no competing interests.

## Authors’ contributions

MBP enrolled the patient, acquired, analyzed and interpreted the patient’s data and was involve in drafting the manuscript; PDF reimplanted the joint prosthesis, reviewed the manuscript for important intellectual content; LP acquired, analyzed, interpreted the patient’s data and reviewed the manuscript for important intellectual content; AM was involved with microbiological investigations, acquired, analyzed, interpreted the patient’s data and reviewed the manuscript for important intellectual content; CP was involved in acquiring the patient’s data and in drafting the manuscript; LM was involved in acquiring the patient’s data and drafting the manuscript; FB reviewed the manuscript and gave the final approval of the version to be published. All authors read and approved the final manuscript.
